# Green Extraction Approaches for Carotenoids and Esters: Characterization of Native Composition from Orange Peel

**DOI:** 10.3390/antiox8120613

**Published:** 2019-12-03

**Authors:** Daniella C. Murador, Fabio Salafia, Mariosimone Zoccali, Paula L. G. Martins, Antônio G. Ferreira, Paola Dugo, Luigi Mondello, Veridiana V. de Rosso, Daniele Giuffrida

**Affiliations:** 1 Bioscience Department, Universidade Federal de São Paulo, Rua Silva Jardin 136, 11015-020 Santos, Brazil; danicarisa@hotmail.com; 2 Department of Chemical, Biological, Pharmaceutical and Environmental Sciences, University of Messina, Polo Annunziata, Viale Annunziata, 98168 Messina, Italy; fsalafia@unime.it (F.S.); pdugo@unime.it (P.D.); lmondello@unime.it (L.M.); 3 Department of Mathematical and Computer Science, Physical Sciences and Earth Sciences, University of Messina, 98168 Messina, Italy; mzoccali@unime.it; 4 Federal Institute of São Paulo, Av. Clara Gianotti de Souza 5180, 11900-000 Registro, Brazil; paula.sfc@gmail.com; 5 Chemistry Department, Federal University of São Carlos, Rodovia Washington Luíz, Km 235, 13565-905 São Carlos, Brazil; giba_04@yahoo.com.br; 6 Chromaleont s.r.l., c/o Department of Chemical, Biological, Pharmaceutical and Environmental Sciences, University of Messina, 98166 Messina, Italy; 7 BeSep s.r.l., c/o Department of Chemical, Biological, Pharmaceutical and Environmental Sciences, University of Messina, 98166 Messina, Italy; 8 Unit of Food Science and Nutrition, Department of Medicine, University Campus Bio-Medico of Rome, 00128 Rome, Italy; 9 Department of Biomedical, Dental, Morphological and Functional Imaging Sciences, University of Messina, Via Consolare Valeria, 98125 Messina, Italy; dgiuffrida@unime.it

**Keywords:** citrus, xanthophylls, esterification, fatty acids, apocarotenoids, ionic liquid, [C_4_mim]Cl, supercritical fluid

## Abstract

Orange peel is a by-product produced in large amounts that acts as a source of natural pigments such as carotenoids. Xanthophylls, the main carotenoid class found in citrus fruit, can be present in its free form or esterified with fatty acids, forming esters. This esterification modifies the compound’s chemical properties, affecting their bioavailability in the human body, and making it important to characterize the native carotenoid composition of food matrices. We aimed to evaluate the non-saponified carotenoid extracts of orange peel (cv. Pera) obtained using alternative green approaches: extraction with ionic liquid (IL), analyzed by high performance liquid chromatography coupled to a diode array detector with atmospheric pressure chemical ionization and mass spectrometry HPLC-DAD-APCI-MS, and supercritical fluid extraction (SFE), followed by supercritical fluid chromatography with atmospheric pressure chemical ionization and triple quadrupole mass spectrometry detection (SFC-APCI/QqQ/MS) in an online system. Both alternative green methods were successfully applied, allowing the total identification of five free carotenoids, one apocarotenoid, seven monoesters, and 11 diesters in the extract obtained with IL and analyzed by HPLC-DAD-APCI-MS, and nine free carotenoids, six carotenoids esters, 19 apocarotenoids, and eight apo-esters with the SFE-SFC-APCI/QqQ/MS approach, including several free apocarotenoids and apocarotenoid esters identified for the first time in oranges, and particularly in the Pera variety, which could be used as a fruit authenticity parameter.

## 1. Introduction

Citrus fruits are among the most produced and consumed fruits worldwide, either fresh or as juice, and especially oranges (*Citrus sinensis* L. Osbeck), which are known for their large consumption and economic importance. Brazil is one of the largest exporters of orange juice around the world; among the most important cultivars in Brazil, the sweet orange cv. Pera has good juice quality and accounts for approximately 30% of the orange crop expected for 2019–2020 [1]. However, this high volume of juice production generates a large amount of waste. The orange peel in turn, can represent an interesting source of carotenoids, which are natural pigments responsible for adding the color to fruits and vegetables, and for providing health-related benefits when ingested by humans [2]. Carotenoid intake is related to improvements in the immune system and reduced risk of developing degenerative diseases, such as cardiovascular diseases, cancer, macular degeneration, and Alzheimer’s disease [3,4,5].

Carotenoids are lipophilic compounds that can be widely classified into two groups: carotenes, which only present carbon and hydrogen atoms in their molecular structures, and xanthophylls, which also contain oxygenated groups, such as hydroxyl, epoxy, and carboxyl groups [6]. The apocarotenoids are a class of carotenoid derivatives that are produced by oxidative cleavages of the parent carotenoid, catalyzed by dioxygenases enzymes of carotenoid cleavage [7,8]. The main class of carotenoids found in oranges is the hydroxylated carotenoids, which include violaxanthin, luteoxanthin, lutein, β-cryptoxanthin, antheraxanthin, mutatoxanthin, and zeaxanthin. These can be presented in their free form or acylated with fatty acids (FAs) in the case of mono- and polyhydroxylated xanthophylls, commonly forming monoesters or diesters [9,10]. In the plant tissues, the esterification helps carotenoid storage and protects sensitive molecules from photo-oxidation [11,12], in addition to increasing the possible structures found in nature, and consequently, the analytical complexity, since a single xanthophyll can be acylated with different FAs [9,13]. For this reason, the carotenoid analysis usually comprises a saponification step in order to simplify the analysis by hydrolyzing the esters and removing interfering compounds; however, saponification may lead to carotenoid degradation and modification, such as isomerization [9,13]. 

Investigations regarding native carotenoid compositions in food matrices, including esters, are mostly lacking in the literature, but this information is highly valuable. Quantifying carotenoid esters is important for better understanding the bioavailability of these compounds in the human body since esterification changes the chemical properties of carotenoids, such as their polarity; esterified xanthophylls are less polar than their corresponding free forms, probably affecting the bioavailability of such compounds [9,14]. Citrus fruits, especially oranges, are well known for being one of the most complex natural source of carotenoids [15], presenting a wide range of esters compositions, which require detailed characterization.

On the other hand, the extraction of carotenoids usually involves the use of conventional organic solvents (COSs) that are highly toxic. The reuse and recycling practices for by-products, such as the orange peel, require urgent attention [16,17] along with an increased focus on sustainability. Therefore, the search for alternative methods for bioactive compound extraction is intensifying. Supercritical fluids and ionic liquids have received attention in the field of chemistry [18], and despite their particularities, all of them have been shown to perform well for extracting bioactive compounds from various matrices [19,20,21,22,23,24]. 

Specifically for matrices rich in carotenoids esters, like oranges, it is crucial to obtain the carotenoids extracts using alternative solvents and to characterize them in their native composition form, to establish associations between all the compounds present in the natural matrix and their benefits to human health. Thus, our aim in this study was to evaluate the native carotenoids composition of orange peel (cv. Pera) using alternative approaches: extraction with ionic liquid (IL), further analyzed by high performance liquid chromatography coupled to a diode array detector with atmospheric pressure chemical ionization and mass spectrometry (HPLC-DAD-APCI-MS), and a supercritical fluid extraction (SFE), followed by supercritical fluid chromatography with atmospheric pressure chemical ionization and triple quadrupole mass spectrometry detection (SFC-APCI/QqQ/MS) in an online system.

## 2. Materials and Methods 

### 2.1. Chemicals

Reagents and solvents of analytical grade used during carotenoid extraction were purchased from Synth (Diadema, Brazil). 1-Methylimidazole and 1-chlorobutane, used for the 1-butyl-3-methylimidazolium chloride ([C_4_mim]Cl) synthesis, were obtained from Sigma-Aldrich (Taufkirchen, Germany). MS-grade methanol (MeOH), methyl tert-butyl ether (MTBE), and water were purchased from Merck (Darmstadt, Germany). The samples were filtered through Millipore (Billerica, MA, USA) membranes (0.22 μm) before HPLC-MS analyses. The standards of (all-*E*)-β-carotene and (all-*E*)-lutein were purchased from Extrasynthese (Genay, France). 

### 2.2. Samples

Oranges (*Citrus sinensis* L. Osbeck) cv. Pera were obtained at a local market in Santos city (São Paulo, Brazil), totalizing a single sample batch of about 10 kg of fruits, further reduced to laboratory samples. The oranges were squeezed in an industrial processor and the peels were processed in a grinder, before being subsequently freeze-dried. Finally, the water free samples were stored at −40 °C in tightly closed packages, until required for analyses.

### 2.3. Obtainment of Carotenoids Extracts

#### 2.3.1. Conventional Extraction with Acetone

A conventional extraction with acetone was performed, since it is one of the most common organic solvents used for carotenoid extraction. Briefly, the carotenoids were manually and exhaustively extracted with acetone from 5.0 g of freeze-dried orange peel, followed by vacuum filtration. The procedure was repeated until the sample became colorless, then, the extract was transferred to diethyl ether/petroleum ether (2:1), washed until acetone free, and concentrated to dryness in a rotary evaporator (temperature (t) < 37 °C) [25,26]. The conventional step of saponification was not applied to enable the analysis of the carotenoids esters present in the extract.

#### 2.3.2. Ionic Liquid Extraction with [C_4_mim]Cl

The carotenoids from orange peel were also extracted using an ionic liquid: 1-butyl-3-methylimidazolium chloride ([C_4_mim]Cl) via ultrasonic-assisted extraction according to Murador et al. [27]. The extraction was performed by mixing 5.0 g of freeze-dried sample, followed by 1:3 solid–liquid ratio (R_(S/L)_) and 1:2 co-solvent ratio (R_(IL/E)_) with ethanol applied as co-solvent. The homogenization was performed with an ultrasound probe (Unique, model DES500, Ultronique, Indaiatuba, Brazil) of 20 kHz and 200 W, at 80% amplitude, under an ice bath, for 5 min, followed by vacuum filtration; the process was repeated six times [27].

#### 2.3.3. Supercritical Fluid Extraction (SFE)

The SFE was performed in a Shimadzu Nexera-UC system (Shimadzu, Kyoto, Japan), combined with a supercritical fluid chromatography (SFC) in one online system. The sample preparation and the SFE conditions were conducted according to Giuffrida et al. [28], with minor modifications. Briefly, the freeze-dried sample of orange peel (10.0 mg) was mixed with 1 g of an adsorbent powder (Miyazaki Hydro-Protect, Patented in Japan No. 3645552) and placed in the extraction vessel of 0.2 mL in the SFE unit (the ID of the extraction chamber was 6 mm and the length was 12 mm), loaded with 100 mg of sample/adsorbent. So, a final amount of 1.0 mg of sample was used, considering the dilution factor. Then, supercritical CO_2_ and methanol were introduced into the vessel, and pressure and temperature conditions of extraction were optimized, as follows: CO_2_ (A) and methanol (B) solvents were used in a gradient as follows: 5% B for 3 min, increasing from 5% to 10% B for 1 min, and changing to 0% B into the SFC analysis mode: flow rate, 2 mL/min; extraction mode, 0–3 min static mode, 3–4 min dynamic mode; extraction vessel temperature, 80 °C; and backpressure regulator, 150 bar [28]. After extraction in the SFE unit, the sample containing CO_2_ was directed to the SFC flow line for chromatographic analysis.

### 2.4. Determination of Native Carotenoids Composition

#### 2.4.1. HPLC-DAD-APCI/MS Analysis 

To characterize the native carotenoid composition in orange peel, the extracts obtained with acetone and with [C_4_mim]Cl were analyzed using a Nexera X2 liquid chromatography system (Shimadzu, Milan, Italy), consisting of a CBM-20A controller, four LC-30AD dual-plunger parallel-flow pumps, a DGU-20 A_5R_ degasser, a CTO-20AC column oven, a SIL-30AC autosampler, and a SPD-M30A diode array detector (DAD). The LC system was coupled to an LCMS-2020 mass spectrometer through an atmospheric pressure chemical ionization (APCI) source (Shimadzu, Kyoto, Japan). 

The HPLC-DAD-MS analysis was performed according to Murillo et al. [29], with some minor modifications. Separation was conducted on a 250 mm × 4.6 mm, 5 μm, YMC C_30_ column. Mobile phases A (MeOH/MTBE/H_2_O, 81:15:4) and B (MeOH/MTBE/H_2_O, 16:80:4), using a linear gradient program, were as follows: 0% B for 20 min; changing from 0% to 100% B in 120 min, returning from 100% to 0% B in 1 min, and maintaining 0% B for 10 min. The flow rate was set to 0.8 mL/min, the injection volume was 20 µL, the column temperature was maintained at 35 °C, the UV-visible (UV-vis) spectra were acquired between 220 and 700 nm, and the chromatograms were processed at 450 nm. Data acquisition was performed using LabSolution software, ver. 5.91 (Shimadzu, Milan, Italy).

The LCMS-2020 detection was achieved using an APCI interface operated in both positive and negative modes; detector voltage, 1.05 kV; interface temperature, 350 °C; DL temperature, 300 °C; heat block temperature, 300 °C; nebulizing gas flow (N_2_), 2.0 L/min; drying gas flow (N_2_), 5.0 L/min; full scan range (positive and negative mode), *m/z* 300 to 1200; and experiment time, 0.2 s. 

The identification of carotenoids and esters was performed using the same tools conventionally applied for carotenoids identification: combined information provided by elution order on C_30_ column, UV-vis spectrum characteristics (maximum absorption wavelength (λ_max_), spectral fine structure (III/II, %), and peak cis intensity (% A_B_/A_II_)), mass spectra, and comparison with data available in the literature [13].

Quantitative data were obtained by HPLC-DAD according to Giuffrida et al. [30]. We used external calibration curves from carotenoid standards of β-carotene, lutein, zeaxanthin, and physalein, at six different concentrations in the range of 0.01 to 100 μg/mL; the esters were quantified using the curves of their correspondents carotenoids. The coefficients of variation (CVs, %) were below 9% in all the HPLC measurements. The carotenoid concentrations are expressed in μg/g of dry matter, and the results are represented as means values of three independent determinations (± standard deviation (SD)).

#### 2.4.2. SFC-APCI/QqQ/MS Analysis

The SFE module was coupled with SFC along with MS in an online system, so the orange peel sample was also analyzed in its entire form in terms of carotenoids composition using a Shimadzu Nexera-UC system (Shimadzu, Kyoto, Japan), consisting of a CBM-20A controller, an SFE-30A module for supercritical fluid extraction, two LC-20AD_XR_ dual-plunger parallel-flow pumps, an LC-30AD_SF_ CO_2_ pump, two SFC-30A back pressure regulators, a DGU degasser, a CTO-20AC column oven, and a SIL-30AC autosampler, also coupled to a LCMS-8050 triple quadrupole (QqQ) mass spectrometer equipped with an APCI source (Shimadzu, Kyoto, Japan). The entire system was controlled by LabSolution ver. 5.8 (Shimadzu, Kyoto, Japan).

The SFC conditions were solvent A (CO_2_) and solvent B (MeOH) in a gradient as follows: 0% B from 4 to 6 min, increasing from 0% to 40% from 6 to 14 min, and maintaining 40% for 2 min [28] at a flow rate of 2.0 mL/min and make-up solvent MeOH at flow 1.0 mL/min [28]. Separations were performed on a partially porous Ascentis Express C_30_, 150 mm × 4.6 mm × 2.7 μm d.p. (MilliporeSigma, St. Louis, MO, USA); the column oven temperature was set to 35 °C and the back pressure regulator to 150 bar. The injection volume for standards was 2 µL. The MS setting followed Giuffrida et al.’s [28] conditions: SCAN (+/–) acquisition mode, selective ion monitoring (SIM) in negative mode, and multiple reaction monitoring (MRM); interface temperature, 350 °C; DL temperature, 200 °C; block heater temperature, 200 °C; nebulizing gas flow (N_2_), 3.0 L/min; drying gas flow (N_2_), 5.0 L/min; full scan range, *m/z* 200 to 1200; and event time, 0.005 s for MRM and SIM and 0.05 s for SCAN. The different apocarotenoids were characterized by SIM and MRM experiments according to the previously optimized transitions determined by product ion scan (PIS) experiments, as reported by Giuffrida et al. [31].

### 2.5. NMR Analysis

NMR analysis was performed to tentatively identify the peak 1 compound, posteriorly shown in the Results and Discussion section. Firstly, the compound was isolated using HPLC-DAD by collecting the fractions in a vial when the compound was observed to elute by chromatogram monitoring at 450 nm. For this, the same YMC C_30_ column was used, but the linear gradient was reduced according to the following conditions: mobile phase A (MeOH/MTBE/H_2_O, 81:15:4) and phase B (MeOH/MTBE/H_2_O, 16:80:4), beginning at 1% B; changing from 1% to 20% B in 5 min; increasing from 20% to 100% B in 2 min; maintaining 100% B for 5 min; returning from 100% to 1% B in 1 min; and remaining at 1% B for 5 min. The HPLC-DAD conditions were also the same: flow rate, 0.8 mL/min; column temperature, 35 °C; and UV-vis spectra acquired between 220 and 700 nm. After the collections, all fractions were joined and completely dried under vacuum and nitrogen, forming a total amount of about 3.4 mg.

All the NMR analyses were performed using 14.1 T (600.23 MHz) equipment for hydrogen frequency, using a TXI cryogenic probe (^1^H/^13^C/^15^N) with a 5 mm internal diameter, maintaining the temperature of 298 K, and using tetramethylsilane (TMS) as the internal reference.

### 2.6. Statistical Analysis

The experiments were conducted in triplicate, and data are expressed as the mean ± standard deviation (SD). The differences between the samples extracted with acetone and [C_4_mim]Cl were detected using a *t*-test; differences were considered significant at *p* < 0.05. The statistical analysis was performed using Microcal Origin 5.0 software (Northampton, MA, USA).

## 3. Results and Discussion

### 3.1. Determination of Native Carotenoids Composition

#### 3.1.1. HPLC-DAD-APCI/MS Analysis 

Despite the scarcity of literature regarding the native carotenoid composition of foods, this type of characterization is crucial for improving our understanding about the bioaccessibility and bioavailability of these compounds and to quantify the natural occurrence of carotenoids and the relationships among them. We used the HPLC-DAD-APCI/MS methodology to tentatively identify the carotenoids and carotenoid esters present in orange peel cv. Pera samples. The presence of FAs complicates chromatography separation; therefore, although several chromatographic conditions and linear gradients of MeOH, MTBE, and H_2_O combinations were tested, some co-elutions were noted. The literature reports that two or more xanthophyll esters almost always co-elute independent of the column and mobile phase [13,32], impairing the identification of some compounds.

The esterification of xanthophylls with FAs does not modify the chromophore or its light absorption properties, so the UV-vis spectrum of the acylated carotenoid is identical to the spectrum of the free compound [33]. Some carotenoids have identical molecular weights, such as free violaxanthin and luteoxanthin, both presenting at *m/z* 601. Therefore, combining the information provided by DAD and MS detectors (UV-vis spectrum and MS (APCI+/–) spectra and fragmentation pattern, respectively) is mandatory for the identification of xanthophyll esters [14,34,35]. 

**Figure 1:**
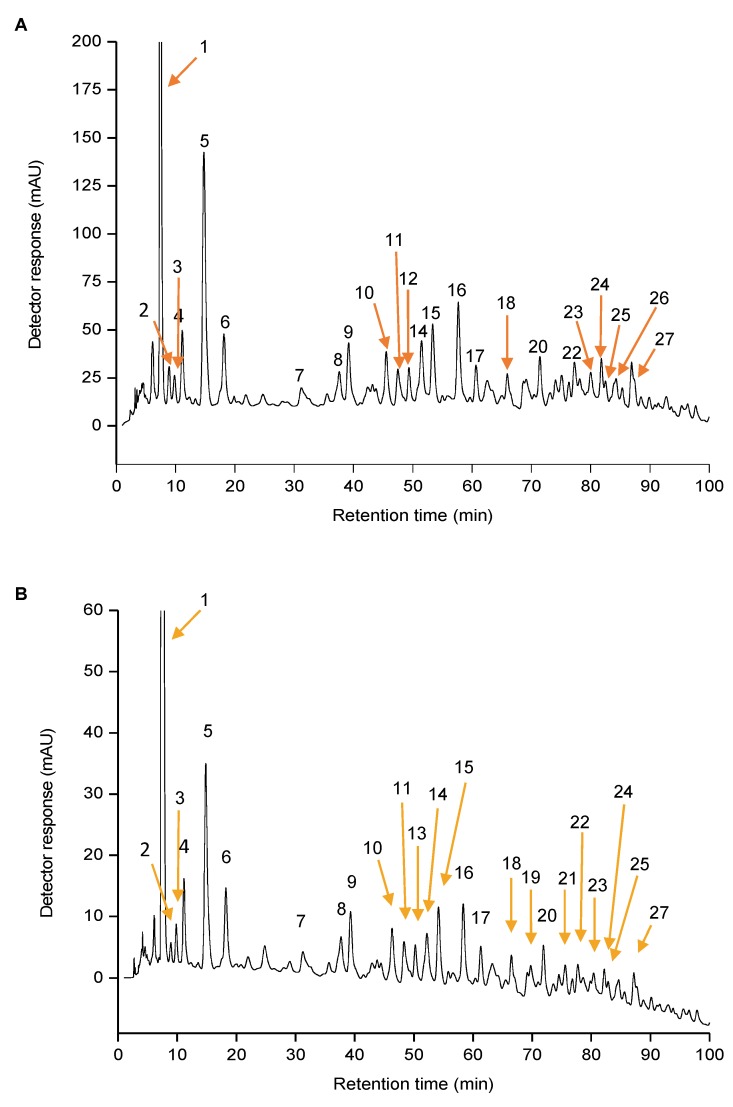
Chromatograms processed at 450 nm, determined using high performance liquid chromatography coupled to diode array detector (HPLC-DAD), of the native carotenoids extracts of orange peel (*Citrus sinensis* L. Osbeck) cv. Pera samples obtained by conventional extraction with (**A**) acetone and (**B**) ionic liquid ([C_4_mim]Cl). The peak characterization is provided in Table 1.

Figure 1 shows the chromatograms obtained by HPLC-DAD using acetone and [C_4_mim]Cl as extracting solvents, and the corresponding chromatographic, UV-vis and MS characteristics of the major carotenoids and esters are shown in Table 1. The major compounds that could be identified in both samples were: (all-*E*)-lutein and (all-*E*)-zeaxanthin (peaks 5 and 6), which were also found in the pulp of the same orange variety (cv. Pera), by Petry and Mercadante [9]. 

**Figure 2:**
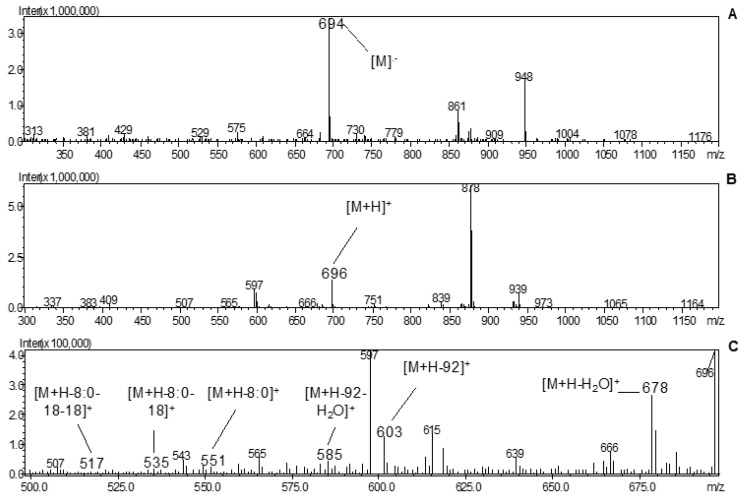
MS spectra of (all-*E*)-lutein 3-*O*-C_8:0_ (peak 11): (**A**) −MS, (**B**) +MS and (**C**) +MS fragmentation.

A total of five free carotenoids (peaks 2, 5, 6, 14, and 16), including one apocarotenoid (peak 4), seven monoesters (peaks 7, 9, 10, 11, 12, 13 and 15), and 11 diesters (17, 18, 19, 20, 21, 22, 23, 24, 25, 26, and 27) were tentatively identified. The compound corresponding to peak 1 was not identified using the present methodology, and is further discussed in Section 3.2. The same free carotenoids profile was found in the samples extracted with acetone and with the ionic liquid, whereas among the esters, some differences were noted. In the sample extracted with acetone (Figure 1A), (9*Z*)-violaxanthin-C_8:0_, (all-*E*)-antheraxanthin-C_10:0_-C_12:0,_ and (all-*E*)-violaxanthin-C_14:0_-C_14:0_ (peaks 13, 19, and 21, respectively) were not detected, whereas in the sample extracted with [C_4_mim]Cl (Figure 2B), only all-*trans*-antheraxanthin-C_12:0_ and all-*trans*-lutein-C_12:0_-C_18:0_ (peaks 12 and 26, respectively) were not determined. 

The carotenoid esters observed were mainly formed from violaxanthin followed by lutein and antheraxanthin esters. Violaxanthin was not found in its free form, but it was the most abundant carotenoid considering both its mono- and diesters. Violaxanthin esters were also always detected in orange pulp from cv. Pera orange [9] and in the juice of different varieties [10,15,36,37]. According to Giuffrida et al. [30], violaxanthin and antheraxanthin and their 5,8-furanoid derivatives are among the major carotenoids present in oranges. β-Cryptoxanthin and its esters, which are also commonly found in oranges [9,10,15,30,36] were not detected using the present methodology. Here, lutein was found either as free carotenoid, or mono- or diester in Pera orange peel, whereas Petry and Mercadante [9] did not detect any ester derivative in the pulp of the same variety, whereas Giuffrida et al. [30] detected both free carotenoid and its diesters in different orange juices varieties. 

We found the xanthophylls acylated mainly with lauric (C_12:0_), myristic (C_14:0_), and palmitic (C_16:0_) acids, in agreement with the findings reported by Petry and Mercadante [9] for the pulp from Pera orange, and also by Dugo et al. [15] and Giuffrida et al. [9,30] for orange juices from various different varieties. According to these findings, these FAs seem to be the most abundant in oranges, but in the present study other esters of short-, medium-, and long-chain FAs were detected, including butyrate (C_4:0_), caproate (C_6:0_), caprylate (C_8:0_), caprate (C_10:0_), and stearate (C_18:0_). 

The detection of the protonated molecule ([M+H]^+^) was noted in most of the compounds, with the exception of (all-*E*)-lutein, which was detected at *m/z* 568 ([M]^−^•) in the APCI negative ionization mode and at *m/z* 551 ([M+H−18]^+^) in the APCI positive ionization mode due to the formation of a stable allylic carbocation; (all-*E*)-antheraxanthin-C_12:0_ and (9*Z*)-violaxanthin-C_4:0_-C_16:0_, which were detected at *m/z* 749 and 891*,* respectively, correspond to the loss of one water molecule from the protonated molecules ([M+H−18]^+^). 

The neutral loss of one water molecule ([M+H−18]^+^) was also detected for both mono- and diesters of violaxanthin. Additionally, in some cases, it was detected also the loss of two water molecules ([M+H−18−18]^+^), and/or a C_7_H_8_ fragment ([M+ H−92]^+^) due to the in-chain loss of toluene alone or along with one water molecule ([M+ H−92−18]^+^). For monoesters, fragments at *m/z* 583 and 565 were detected, corresponding to elimination of the fatty acid moiety ([M+H−FA]^+^) alone and together with one water molecule ([M+ H−FA−18]^+^), respectively. For the violaxanthin diesters, two fragments at *m/z* 565 and 547 were detected due the loss of two FA moieties ([M+ H−FA1−FA2]^+^) and along with a water molecule ([M+H−FA1−FA2−18]^+^), respectively. These findings agree with the data reported by Petry and Mercadante [9], Giuffrida et al. [10], and Dugo et al. [14,38] related to orange pulp and juice from different varieties. In the present study, most of the violaxanthin esters presented in their (*Z*)-isomeric configuration (eight out of nine), which were identified considering the corresponding UV-vis spectra characteristics and elution order. 

The free form of lutein (peak 5) was found to be the main carotenoid in all the samples analyzed, and five more (all-*E*)-lutein esters (peaks 7, 10, 11, 25, and 26) were identified. As well as occurs with all esters, mono- and diesters derived from lutein present the same UV-vis spectrum from its free from, since the acylation of one or two FAs to the hydroxyl groups in the terminal rings does not alter the chromophore [32]. This characteristic allowed the identification and differentiation of the esters derived from lutein and zeaxanthin considering their different UV-vis spectra; free (all-*E*)-zeaxanthin (peak 6) was also found to be one of the main carotenoids, and its esters exhibit the same molecular weight as lutein, but different UV-vis spectra. Lutein has an asymmetric structure, containing one β- and one *ε*-ring, so regioisomers could be formed depending on whether the ester is monoacylated with one FA or diacylated with two different FAs (heterodiester) [9]. In APCI (+)-MS conditions, it is possible to determine if the hydroxyl group or the acylated FA is present at position 3′-*O* or 3-*O*; for monoesters, the lutein 3′-*O*-monoester shows in-source fragments [M+ H−FA]^+^ at *m/z* 551 as the most abundant ion, whereas [M+H−H_2_O]^+^ at higher *m/z* represents the predominant positive fragment ion of 3-*O*-monoester, for example, at *m/z* 678 for (all-*E*)-lutein 3-*O*-C_8:0_, as shown in Figure 2. For heterodiesters, the most abundant in-source fragment ion indicates the loss of the FA located at position 3′ (ε-ring); consequently, the FA attached to position 3 (β-ring) can be deduced by exclusion [39,40]. However, for the diesters, we observed no perceptible differences in MS spectra between regioisomeric forms of (all-*E*)-lutein C_12:0_-C_18:0_ and (all-*E*)-lutein C_14:0_- C_16:0_ (peaks 25 and 26, respectively). 

In relation to the fragmentation pattern of antheraxanthin esters, we again detected the neutral loss of one water molecule from the protonated molecule ([M+H−H_2_O]^+^), and fragments indicating the loss of C_7_H_8_ (toluene) ([M+H−92]^+^) were detected for both mono- and diesters, in agreement with the report by Petry and Mercadante [9] in Pera orange pulp. Fragments at *m/z* 567, representing the loss of FA ([M+H−FA]^+^), were found in both monoesters (peaks 12 and 15), and also fragments at *m/z* 549 and 531 were detected, corresponding to the losses of FA along with one ([M+H−FA−18]^+^) and two ([M+ H−FA−18−18]^+^) water molecules, respectively (peaks 15 and 12, respectively). The fragments at *m/z* 567 and 549 in antheraxanthin monoester were also reported by Dugo et al. [38], Giuffrida et al. [10], and Petry and Mercadante [9]. Regarding antheraxanthin diesters, the fragment representing the carotenoid backbone at *m/z* 549 after the loss of two FA moieties was detected; in this case, a heterodiester ([M+H−FA1−FA2]^+^) was detected by peak 19 and at *m/z* 531 due the elimination of one water molecule, along with FAs and, a homodiester ([M+H−2FA−18]^+^) detected by peak 18. Antheraxanthin diesters, including their fragments, were also found by Petry and Mercadante [9].

The quantitative data regarding the carotenoids and esters amounts were calculated using external standard calibration curves [10]. As peak 1 had not yet been identified, it was not considered in the quantification because it was abundant and would have interfered with the statistical analysis. The results are shown in Table 2, and they represent the average of three independent experiments. 

Considering the total carotenoid content, the application of acetone and [C_4_mim]Cl did not lead to significant differences in the total carotenoid amount: 97.4 ± 17.1 and 64.2 ± 9.3 µg/g of dry matter, respectively (*p* = 0.07). Free carotenoids were the main compounds found in all the samples (varying from 49.9% to 52.2%), but in this case, extraction with acetone resulted in a significantly higher content of free carotenoids than with [C_4_mim]Cl (*p* < 0.05). For the monoesters, the different extractions did not result in significant differences in the extracted amount (*p* = 0.49), whereas the use of acetone led to an increased content of diesters compared with [C_4_mim]Cl (*p* < 0.05).

#### 3.1.2. SFE-SFC-APCI/QqQ/MS Analysis

As previously stated, the use of supercritical fluids as the extracting phase for bioactive compounds is considered a promising approach in the green chemistry field. Different fluids can be used such as carbon dioxide, ethane, propane, butane, pentane, ethylene, ammonia, sulfur dioxide, water, or chlorodifluoromethane [41]. The literature reports carbon dioxide (CO_2_) as one of the most widely used supercritical fluids, which is especially convenient for carotenoid extraction; it is the most preferred in the pharmaceuticals and food industries due to its relatively low critical properties, making it ideal for thermally labile components, such as the carotenoids [41,42].

Supercritical fluids have been used for both carotenoids extraction (SFE) and separation (SFC) [43,44,45,46]. However, only recently was the direct online extraction and determination of carotenoids using a SFE-SFC/MS methodology reported [47]. The SFE-SFC-APCI/QqQ/MS online system has already been used to determine carotenoids and apocarotenoids from habanero pepper (*Capsicum chinense* Jacq.) [31,47], tamarillo fruit (*Solanum betaceum* Cav.) [28], different microalgae strains [48], and human blood samples [49]. This system involves the use of supercritical CO_2_, a small amount of MeOH compared to other analytical systems, and its execution time is short [43,47]. 

In the present work, freeze-dried samples of orange peel (cv. Pera) were used in the SFE, which lasted a total of four minutes, followed by the SFC analysis that was performed in 12 min; so, the final result generated from the whole process was achieved in 16 min. This methodology can be considered innovative compared with traditional solid−liquid extraction and conventional liquid chromatography, which require a much longer analytical time and use more solvent, and this method is fully automated, reducing operator error and analyte losses [43]. 

**Figure 3:**
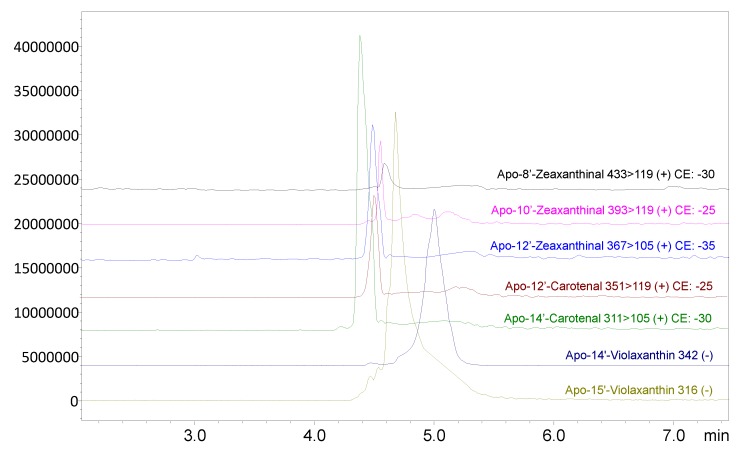
The enlargements of the ion chromatogram obtained in SIM and MRM modes relative to some detected apocarotenoids obtained through SFE-SFC-APCI(+/−)/QqQ/MS analysis. The optimized transitions for each apocarotenoid are shown for both the quantifier (Q) and the qualifier (q) ions, with the relative used collision energies (CEs).

According to the results from the SFE-SFC/MS analysis, we detected nine free carotenoids, six carotenoids esters, 19 apocarotenoids, and eight apo-esters, which were identified using the available standard, the compounds’ elution order, along with a full scan, selected ion monitoring (SIM), and multiple reaction monitoring (MRM) experiments. The most abundant compounds found were β-citraurinol and apo-14′-violaxanthinal. Table 3 lists the overall carotenoids, apocarotenoids, and their esters detected in orange peel by SFE-SFC-APCI/QqQ/MS analysis, and Figure 3 depicts the corresponding enlargements of the ion chromatogram obtained in SIM and MRM modes relative to some detected apocarotenoids.

Through the use of this methodology, many apocarotenoids were detected compared to the HPLC analysis. This highlights that different methods of analysis may provide different compounds profiles even with the same matrix. 

Apocarotenoids, in general, are still poorly reported in the literature, and normally analyzed using conventional liquid chromatography. Chedea et al. [50] found 10′-apo-β-caroten-10′-ol, 10′-apo-β-caroten-10′-al, 8′-apo-β-caroten-8′-ol, 8′-apo-β-caroten-8′-al, and 6′-apo-β-caroten-6′-ol as the main compounds in the orange peel of the Valencia variety, detected by HPLC; in the Navel variety, β-cryptoxanthin was the main carotenoid. However, as already discussed, the SFE-SFC approach is an advanced analytical methodology, especially considering the possible online coupling to a DAD and MS detectors. To the best of our knowledge, this was the first attempt to analyze an orange peel sample using the SFE-SFC-APCI/QqQ/MS system, resulting in the most detailed apocarotenoids and apocarotenoid esters characterization in oranges, and in particular in the Pera variety, which could be also used as a fruit authenticity parameter.

### 3.2. NMR Analysis

**Figure 4:**
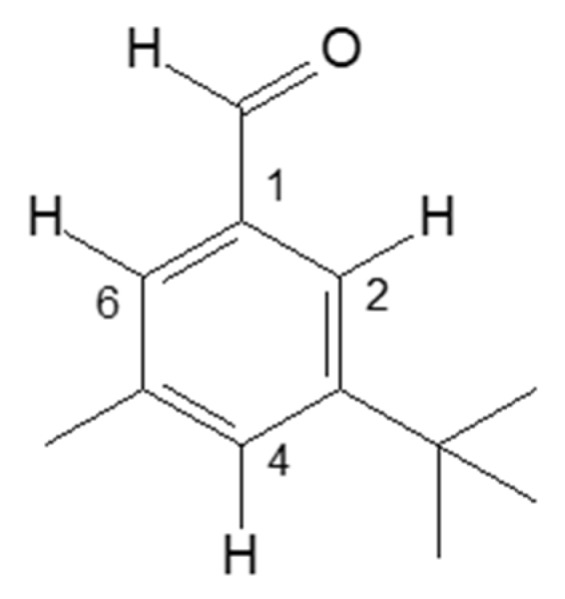
Chemical structure proposed for the compound named as peak 1, detected in the non-saponified carotenoids extract of orange peel (*C. sinensis* L. Osbeck) cv. ‘Pera’, named: structure 1. N^o^ 1, 2, 4 and 6 are carbon number.

According to the chromatographic, UV/vis and mass spectroscopy characteristics provided by the HPLC-DAD-APCI/MS analysis, it was not possible to identify the compound named as peak 1. Therefore, as previously described, the compound was isolated, and a NMR analysis was carried out. Figure 4 presents the chemical structure proposed for the unidentified compound from the NMR analysis, and Table 4 shows its δ chemical displacement (ppm) and J coupling constant (Hz). However, considering the information provided by all the analyses, the NMR data did not match with the UV-vis and MS data obtained by the HPLC-DAD-APCI/MS analysis and reported in Table 1; therefore, unfortunately, it was not possible to identify or to elucidate the structure of compound named as 1. Despite that, the authors strongly suggest that the compound might be the result of some degradation taking place.

**Figure 5:**
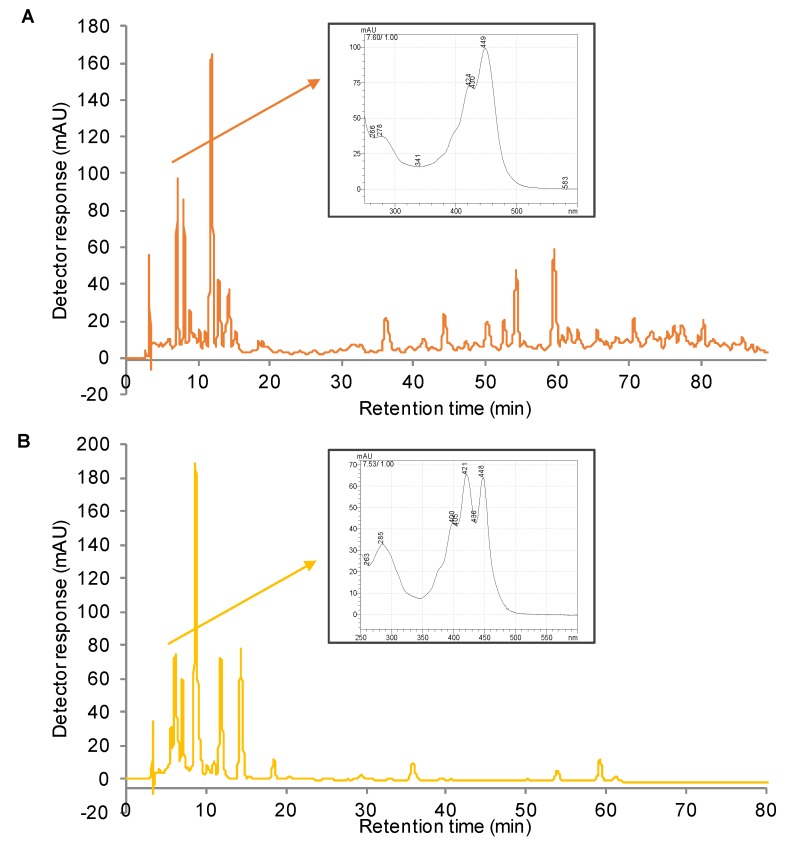
Chromatograms processed at 450 nm, obtained by HPLC-DAD, of the native carotenoids extract (non-saponified) (**A**), and the saponified carotenoids extract (**B**) of orange peel *(C. sinensis* L. Osbeck) cv. ‘Pera’. Inserts: UV-vis spectra of compounds eluted at the same retention time and in the same chromatographic conditions, relative respectively to the unidentified compound found in the non-saponified extract (**A**), and to the identified (all-*E*)-luteoxanthin detected in the saponified extract (**B**). The chromatographic conditions were the same reported for the HPLC-DAD-APCI/MS analysis.

Figure 5 shows a comparison between the chromatograms of the native carotenoids extract (non-saponified), and the saponified carotenoids extract, obtained by HPLC-DAD, under the same analitical conditions. At the same retention time (range 7.5–7.6 min), the unidentified compound named as 1, was detected in the non-saponified sample, while in the saponified one, it was not noted; the peak eluted at 7.53 min presented a UV/vis spectra probably correspondent to (all-*E*)-luteoxanthin, previously identified by Murador et al. [27], in the same saponified orange extract.

It is well known that carotenoids are highly susceptible to degradation when exposed to heat, low pH, and light, promoting the rearrangement or formation of degradation compounds, such as cis-isomers, epoxides, short chain products, and volatile compounds [51,52]. The organic acids liberated during the processing of the citrus fruit, in this case, that could be promoted by the extraction process itself, can be strong enough to promote these modifications [51,53]. In the saponification step, the KOH neutralizes the acids present, however, in non-saponified samples, they remain in the extracts. The mechanisms involved in the degradation pathways are complex, and not well elucidated, but it is known that acids are able to initiate the carotenoids degradation in food matrices [51,54], which probably has occurred in the present study, resulting in the unidentified product here reported.

## 4. Conclusions

The concern about sustainability and environmental issues has been growing around the world, and the search for alternative methods for extracting bioactive compounds with reduced impacts is increasing. In this paper, we reported the application of two extraction methodologies based on sustainable approaches for the characterization of the native carotenoid composition in orange peel (*C. sinensis* L. Osbeck) cv. Pera, resulting in a total identification of 10 free carotenoids, 12 monoesters, 11 diesters, 20 apocarotenoids, and eight apo-esters, considering both methods. The present study presents an extensive and complete characterization about the native carotenoids composition from orange peel, supporting and encouraging future studies regarding the bioavailability of carotenoids and esters, to better understand how the esterification affects the biological effects of these compounds. 

## Figures and Tables

**Table 1:** Chromatographic, UV-visible (UV-vis) and mass spectroscopic characteristics of the carotenoids identified in the native carotenoids extracts of orange peel, obtained by high performance liquid chromatography coupled to diode array detector with atmospheric pressure chemical ionization and mass spectrometry (HPLC-DAD-APCI/MS) analyses.

Peak	Carotenoid	*t*_R_^a^ (min)	*λ_max_*^b^ (nm)	% III/II	% *A*_B_/*A*_II_	[M+H]^+^ (*m/z*)	[M]^−^• (*m/z*)	Fragment Ions (*m/z*)
1	n.i.	7.4–8.2	451	n.c.	0	435	434	419, 362, 391
2	(all-*E*)-luteoxanthin	8.9–10.0	399, 422, 449	n.c.	n.d.	601	n.d.	583[M+H−18]^+^, 509[M+H−18−18]^+^, 565[M+H−92]^+^
3	n.i.	9.8–11.5	463	n.c.	0	589	n.d.	571 [M+H−18]^+^, 553 [M+H−18−18]^+^
4	sintaxanthin	11.1–12.8	417, 439, 468	50	0	430	429	412[M+H−18]^+^, 394[M+H−18−18]^+^, 338[M+H−92]^+^
5	(all-*E*)-lutein	14.8–17.5	423, 444, 472	56	0	551	568	551[M+H−18]^+^
6	(all-*E*)-zeaxanthin	18.2–21.7	423, 450, 476	14	0	569	568	551[M+H−18]^+^
7	(all-*E*)-lutein 3-*O*-C_4:0_	31.2–36.3	418, 440, 470	n.c.	0	638	636	620[M+H−18]^+^, 602[M+H−18−18]^+^, 546[M+H−92]^+^, 551[M+H−4:0]^+^, 533[M+H−4:0−18]^+^, 514[M+H−4:0−18−18]^+^, 510[M+H−92−18−18]^+^
8	n.i.	37.6–42.1	447	33	n.d.	712	710	694 [M+H−18]^+^, 676 [M+H−18−18]^+^, 620 [M+H−92]^+^
9	(13*Z*)- or (15*Z*)-violaxanthin-C_12:0_	39.2–44.0	329, 418, 438, 468	n.c.	n.c.	783	n.d.	765[M+H−18]^+^, 747[M+H−18−18]^+^, 673[M+H−92−18]^+^, 583[M+H−12:0]^+^, 565[M+H−12:0−18]^+^, 547[M+H−12:0−18−18]^+^
10	(all-*E*)-lutein 3-*O*-C_6:0_	45.5–50.7	416, 436, 465	n.c.	n.c.	666	664	648[M+H−18]^+^, 630[M+H−18−18]^+^, 573[M+H−92]^+^, 556[M+H−92−18]^+^, 551[M+H−6:0]^+^, 538[M+H−92−18−18]^+^, 533[M+H−6:0−18]^+^
11	(all-*E*)-lutein 3-*O*-C_8:0_	47.5–52.8	419, 441, 470	n.c.	0	696	694	678[M+H−18]^+^, 603[M+H−92]^+^, 585[M+H−92−18]^+^, 551[M+H−8:0]^+^, 533[M+H−8:0−18]^+^, 517[M+H−8:0−18−18]^+^
12	(all-*E*)-antheraxanthin-C_12:0_	49.3–54.4	417, 436, 468	n.c.	0	n.d.	n.d.	749[M+H−18]^+^, 675[M+H−92]^+^, 567[M+H−12:0]^+^, 531[M+H−12:0−18−18]^+^
13	(9*Z*)-violaxanthin-C_8:0_	50.3–54.3	328, 417, 436, 466	n.c	18	728	726	710[M+H−18]^+^, 692[M+H−18−18]^+^, 636[M+H−92]^+^, 600[M+H−92−18−18]^+^, 583[M+H−8:0]^+^, 565[M+H−8:0−18]^+^, 548[M+H−8:0−18−18]^+^
14	(all-*E*)-α-carotene	51.5–56.6	418, 446, 470	n.c.	0	537	536	n.d.
15	(*Z*)-antheraxanthin-C_18:0_	53.4–58.1	330, 416, 436, 467	n.c.	n.c.	851	n.d.	833[M+H−18]^+^, 759[M+H−92]^+^, 741[M+H−92−18]^+^, 567[M+H−18:0]^+^, 549[M+H−18:0−18]^+^, 475[M+H−18:0−92]^+^
16	(all-*E*)-β-carotene	57.7–62.4	426, 450, 478	0	0	537	536	444[M+H−92]^+^
17	(9*Z*)-violaxanthin-C_4:0_-C_16:0_	60.7–65.2	328, 415, 439, 466	59	n.c.	n.d.	n.d.	891[M+H−18]^+^, 873[M+H−18−18]^+^, 821[M+H−4:0]^+^, 653[M+H−16:0]^+^, 565[M+H−4:0−16:0]^+^
18	(13*Z*)- or (15*Z*)-antheraxanthin-C_12:0_-C_12:0_	65.9–69.9	329, 416, 437, 466	n.c.	n.c.	949	n.d.	913[M+H−18−18]^+^, 857[M+H−92]^+^, 749[M+H−12:0]^+^, 531[M+H−12:0−12:0−18]^+^
19	(all-*E*)-antheraxanthin-C_10:0_-C_12:0_	69.8	415, 441, 469	60	0	921	n.d.	903[M+H−18]^+^, 885[M+H−18−18]^+^, 829[M+H−92]^+^, 811[M+H−92−18]^+^, 749[M+H−10:0]^+^, 731[M+H−10:0−18]^+^, 721[M+H−12:0]^+^, 703[M+H−12:0−18]^+^, 549[M+H−10:0−12:0]^+^
20	(9*Z*)-violaxanthin-C_12:0_-C_14:0_	71.4–74.9	330, 418, 437, 466	84	12	993	n.d.	975[M+H−18]^+^, 883[M+H−92−18]^+^, 793[M+H−12:0]^+^, 775[M+H−12:0−18]^+^, 765[M+H−14:0]^+^, 747[M+H−14:0−18]^+^, 565[M+H−12:0−14:0]^+^
21	(all-*E*)-violaxanthin-C_14:0_-C_14:0_	75.2–75.6	419, 443, 471	42	0	1022	n.d.	1004[M+H−18]^+^, 986[M+H−18−18]^+^, 930[M+H−92]^+^, 794[M+H−14:0]^+^, 565[M+H−14:0−14:0]^+^, 547[M+H−14:0−14:0−18]^+^
22	(9*Z*)-violaxanthin-C_14:0_-C_14:0_	77.3–80.4	330, 415, 438, 466	87	21	1022	n.d.	1004[M+H−18]^+^, 912[M+H−92−18]^+^, 776[M+H−14:0−18]^+^, 565[M+H−14:0−14:0]^+^, 547[M+H−14:0−14:0−18]^+^
23	(13*Z*)- or (15*Z*)-violaxanthin C_12:0_-C_18:0_	80.1–80.3	330, 417, 440, 469	n.c.	18	1050	n.d.	1031[M+H−18]^+^, 959[M+H−92]^+^, 939[M+H−92−18]^+^, 850[M+H−12:0]^+^, 766[M+H−18:0]^+^, 565[M+H−12:0−18:0]^+^, 547[M+H−12:0−18:0−18]^+^
24	(13*Z*)- or (15*Z*)-violaxanthin C_14:0_-C_16:0_	81.8–84.5	330, 415, 438, 467	86	16	1050	n.d.	1031[M+H−18]^+^, 959[M+H−92]^+^, 939[M+H−92−18]^+^, 821[M+H−14:0]^+^, 803[M+H−14:0−18]^+^, 793[M+H−16:0]^+^, 775[M+H−16:0−18]^+^, 565[M+H−14:0−16:0]^+^, 547[M+H−14:0−16:0−18]^+^
25	(all-*E*)-lutein 3-*O*-C_12:0_- 3′-*O*-C_18:0_ or 3′-*O*-C_12:0_- 3-*O*-C_18:0_	82.6–82.8	420, 446, 469	29	0	1016	n.d.	998[M+H−18]^+^, 816[M+H−12:0]^+^, 732[M+H−18:0]^+^, 533[M+H−12:0−18:0]^+^
26	(all-*E*)-lutein 3-*O*-C_14:0_- 3′-*O*-C_16:0_ or 3′-*O*-C_14:0_- 3-*O*-C_16:0_	83.3	420, 444, 469	33	0	1016	n.d.	998[M+H−18]^+^, 788[M+H−14:0]^+^, 760[M+H−16:0]^+^, 533[M+H−14:0−16:0]^+^
27	(13*Z*)- or (15*Z*)-violaxanthin-C_16:0_-C_16:0_	85.4–85.6	331, 414, 439, 467	n.c.	n.c.	1077	n.d.	1059[M+H−18]^+^, 821[M+H−16:0]^+^, 803[M+H−16:0−18]^+^, 565[M+H−16:0−16:0]^+^

^a^ Retention time on the C_30_ column; ^b^ Linear gradient of methanol/MTBE/water; λ_max_, maximum absorption wavelength (nm); % III/II, spectral fine structure; % *A*_B_/*A*_II_, intensity of *cis* peak; n.i., not identified; n.d., not detected; n.c., not calculated.

**Table 2:** Content of free carotenoids, monoesters, diesters, and total carotenoids of the extracts obtained with acetone and with ionic liquid ([C_4_mim]Cl) from orange peel (*Citrus sinensis* L. Osbeck) cv. Pera.

Type of Extract	Quantification of Carotenoids and Esters (µg/g of Dry Matter) *			
	Free Carotenoids	Monoesters	Diesters	Total Carotenoids
Acetone	50.9 ± 6.2 ^a^	29.3 ± 10.0 ^a^	20.3 ± 5.9 ^a^	97.4 ± 17.1 ^a^
[C_4_mim]Cl	32.1 ± 6.2 ^b^	24.6 ± 3.8 ^a^	7.6 ± 1.8 ^b^	64.2 ± 9.3 ^a^

* Values are expressed as the mean ± standard deviation (SD) (*n* = 3 independent experiments). Means in the same column followed by different letters (a,b) differed significantly (*p* < 0.05).

**Table 3:** Overall carotenoids, apocarotenoids, and their esters detected by supercritical fluid extraction and supercritical fluid chromatography with atmospheric pressure chemical ionization and triple quadrupole mass spectrometry detection (SFE-SFC-APCI (+/–)/QqQ/MS) analysis in orange peel with relative selected ion monitoring (SIM) *m/z* values, multiple reaction monitoring (MRM) with quantifier (Q) and qualifier (q) transitions, collision energy (CE, in volts), and ion ratio (%).

Compounds	SIM (−) *m/z*	MRM Transition (CE)		Ion Ratio % (+)
		Quantifier (Q)	Qualifier (q)	
**Free Carotenoids**				
Luteoxanthin	600	n.d.	n.d.	n.c.
Antheraxanthin	478	n.d.	n.d.	n.c.
Lutein	568	n.d.	n.d.	n.c.
Zeaxanthin	568	+ 569 > 119 (−39)	+ 569 > 135 (−22)	95
β-cryptoxanthin	552	+ 553 > 119 (−32)	+ 553 > 145 (-38)	61
Phytoene	544	n.d.	n.d.	n.c.
β-carotene	536	+ 537 > 119 (−39)	+ 537 > 121 (−32)	84
β-cryptoxanthin-5,6-epoxide	568	n.d.	n.d.	n.c.
β-carotene-5,6-epoxide or β-carotene-5,8-epoxide	552	n.d.	n.d.	n.c.
**Carotenoids Esters**				
antheraxanthin-C12:0	766	n.d.	n.d.	n.c.
zeaxanthin-C12:0	750	n.d.	n.d.	n.c.
lutein-C14:0 or zeaxanthin-C14:0	778	n.d.	n.d.	n.c.
β-cryptoxanthin-C12:0	734	n.d.	n.d.	n.c.
β-cryptoxanthin-C14:0	762	n.d.	n.d.	n.c.
β-cryptoxanthin-C16:0	790	n.d.	n.d.	n.c.
**Apo-Carotenoids**				
β-citraurinol	434	n.d.	n.d.	n.c.
β-apo-8′-carotenal	416	+ 417 > 119 (−25)	+ 417 > 105 (−35)	73
β-apo-10′-carotenal	376	+ 377 > 105 (−35)	+ 377 > 119 (−30)	79
β-apo-12′-carotenal	350	+ 351 > 105 (−35)	+ 351 > 119 (−25)	74
β-apo-14′-carotenal	310	+ 311 > 105 (−25)	+ 311 > 119 (−25)	77
apo-8′-zeaxanthinal	432	+ 433 > 119 (−30)	+ 433 > 105 (−35)	95
apo-10′-zeaxanthinal	392	+ 393 > 105 (−35)	+ 393 > 119 (−25)	92
apo-12′-zeaxanthinal	366	+ 367 > 105 (−35)	+ 367 > 119 (−30)	80
apo-14′-zeaxanthinal	326	+ 327 > 105 (−35)	+ 327 > 119 (−30)	61
apo-15-zeaxanthinal	300	+ 301 > 173 (−15)	+ 301 > 105 (−30)	57
apo-8-luteinal	432	+ 415 > 119 (−40)	+ 415 > 91 (−50)	95
apo-10-luteinal	392	+ 375 > 105 (−40)	+ 375 > 91 (−50)	91
apo-12-luteinal	366	+ 349 > 105 (−40)	+ 349 > 91 (−50)	90
apo-14-luteinal	326	+ 309 > 91 (−50)	+ 309 > 105 (−40)	55
apo-8′-violaxanthinal	448	n.d.	n.d.	n.c.
apo-10′-violaxanthinal	408	n.d.	n.d.	n.c.
apo-12′-violaxanthinal	382	n.d.	n.d.	n.c.
apo-14′-violaxanthinal	342	n.d.	n.d.	n.c.
apo-15′-violaxanthinal	316	n.d.	n.d.	n.c.
**Apo-Esters**				
apo-10′-zeaxanthinal-C4:0	462	+ 463 > 105 (−40)	+ 463 > 119 (−35)	71
apo-10′-zeaxanthinal-C10:0	546	+ 547 > 105 (−35)	+ 547 > 119 (−30)	87
apo-10′-zeaxanthinal-C12:0	574	+ 575 > 105 (−35)	+ 575 > 119 (−30)	75
apo-10′-zeaxanthinal-C14:0	602	+ 603 > 105 (−40)	+ 603 > 119 (−30)	77
apo-8′-zeaxanthinal-C6:0	530	+ 531 > 119 (−40)	+ 531 > 105 (−40)	78
apo-8′-zeaxanthinal-C8:0	558	+ 559 > 105 (−40)	+ 559 > 119 (−40)	70
apo-8′-zeaxanthinal-C10:0	586	+ 587 > 119 (−40)	+ 587 > 105 (−40)	81
apo-8′-zeaxanthinal-C12:0	614	+ 615 > 105 (−40)	+ 615 > 119 (−40)	79

Underlined compounds were the most abundant. n.d., not detected; n.c., not calculated.

**Table 4:** δ chemical displacement (ppm) and J coupling constant (Hz) for the substance from structure 1.

No.	^1^H	^13^C	HMBC
1	-	135.1	-
2	7,46 d (J = 2,40) 1H	123.5	34.8; 123.5; 133.3; 149.1 and 185.3
3	-	149.1 *	-
4	7.18 bs 1H	132.5	123.5; 133.3 and 135.1
5	-	148.8 *	-
6	6.96 d (J = 2,40) 1H	133.3	34.8; 123.5; 132.5; 135.1; 149.1 and 185.4
5-CH_3_	1.31 s 9H	28.7	28.7; 34.8; 123.5 and 149.1
3-CH_3_	1.28 s 9H	28.7	28.7; 34.4; 133.3; 148.8 and 185.3
5 > C<	-	34.8 *	-
3 > C<	-	34.4 *	-

* interchangeable values; d: doublet; s: singlet; bs: broad singlet. HMBC: Heteronuclear Multiple-Bond correlation.
